# Supplementary material for: Clinical and Sociodemographic Profile of Psychomotor Agitation in Mental Health Hospitalisation: A Multicentre Study
Source: Int J Environ Res Public Health. 2022 Nov 30;19(23):15972. doi: 10.3390/ijerph192315972 (PMC9735933; doi:10.3390/ijerph192315972)
Supplement: Supplementary file 1 [file ijerph-19-15972-s001.zip › ijerph-1969981-supplementary.pdf]

International Journal of
Environmental Research and Public Health

MDPI

Supplementary Materia

Table S1. Items of the Spanish version of the Corrigan's ABS scale.

Item	Mean	SD±	Asymmetry	Kurtosis
1. Poor attention paid, easily distracted, inability to concentrate	2.8	0.8	−0.3	−0.3
2. Impulsiveness, impatience, poor tolerance for pain and frustration	3.2	0.8	−1	0.4
3. Little cooperation, does not allow care to be administered, demanding	3.1	0.8	−0.6	−0.2
4. Is violent, threatens people	2.9	1	−0.5	−0.8
5. Explosive or unpredictable outbursts of anger	2.9	0.9	−0.6	−0.6
6. Rocks, rubs, groans, or exhibits other self−stimulating behaviour	2.2	1.1	0.3	−1.2
7. Pulls objects or ties from the bed	1.7	1	1	−0.4
8. Roams around treatment áreas	2.5	1.1	−0.1	−1.3
9. Restlessness which comes and goes, excessive moving	2.8	0.9	−0.5	−0.5
10. Repetitive motor or verbal behaviours	2.7	0.9	−0.3	−0.8
11. Speaks fast, loudly, or excessively	2.9	1	−0.5	−0.9
12. Sudden mood changes	2.8	1	−0.6	−0.7
13. Cries or laughs easily and excessively	2	1	0.6	−0.8
14. Is harmful or insulting	2.7	1	−0.3	−1.1
